# Is anemia still a problem in cART era: a single center study?

**DOI:** 10.1186/1471-2334-14-S2-P48

**Published:** 2014-05-23

**Authors:** P Genet, J Gerbe, V Masse, D Chaoui, B Wifaq

**Affiliations:** 1 Victor Dupouy Hospital, Argenteuil, France

## Aim of the study

In the pre-cART era, anemia was a frequent complication observed in HIV patients. But, recent studies showed a persistent high prevalence of anemia, integrating anemia in the premature aging observed in HIV patients. The aim of the study was to estimate the prevalence of anemia in our patient’s cohort.

## Materials and methods

All patients, except pregnant women, seen in our unit after 2007 were included in this analysis. For each patient, last hemoglobin’s concentration was collected. Incidence of anemia was calculated according WHO’s definition.

## Results

544 patients (260 female, 284 men) with a median age of 45.9 years (18.9-80.8) were included. 513 patients were on ART (94.3%). Median CD4 was 546.5 (14-2707), viral load (VL) < 50 in 75.9% of patients.

Anemia was present in 141 patients (25.9%): mild 18.9%, moderate 6.8%, severe 0.2%. No correlation with VL was seen: mean Hb for patients with CV>50 versus <50 was respectively 13.3 and 13.4. No correlation with CD4 was seen: mean CD4 for anemic and non-anemic patients was respectively 502 and 544 (NS). However, a correlation with age was seen with an incidence of anemia of 24.5% for patients under 60 y and 36.4% for patients ≥60 y (p<0.001). These impact on age was only seen in men with an incidence of anemia of 12.6% for age under 60 y vs 33.3% for age ≥60 y (p<0.001). For women, age has no impact on prevalence of anemia.

## Conclusion

In the era of c-ART, incidence of anemia remains high especially in patients above 60 years. Given the growing age of HIV patients, physicians should be attentive to this problem in the oldest patients. Further prospective studies are needed to better characterize the mechanisms of anemia observed in this population.

